# Medical dissertation basics: analysis of a course of study for medical students

**DOI:** 10.3205/zma001547

**Published:** 2022-04-14

**Authors:** Sophia Griegel, Michael Kühl, Achim Schneider, Susanne J. Kühl

**Affiliations:** 1 University of Ulm, Medical Faculty, Institute for Biochemistry and Molecular Biology, Ulm, Germany; 2 University of Ulm, Medical Faculty, Office of the Dean of Studies, Ulm, Germany

**Keywords:** dissertation, doctoral thesis, scientific curriculum, doctoral supervision, scientific competency development, online teaching

## Abstract

**Background:**

Although the majority of medical students in Germany pursue a doctorate, only a portion of them receive a standardized scientific training, which is reflected in the quality issues seen in medical doctoral theses. The course Medical Dissertation Basics was conceptualized and scientifically monitored in order to support medical doctoral students on the one hand and to improve the quality of their scientific work on the other.

**Methodology:**

The course consists of three modules. Module I, which is an introductory module, covers time and writing management and addresses how to approach literature and the principles of scientific work as well as the chapters required in a dissertation and the dissertation presentation and defense. In the practical module II, doctoral students write sections of their dissertation chapters and receive feedback via peer and expert reviews. Module III includes training on dissertation presentations and their defense. For objective analysis purposes, a multiple-choice test was administered before and after module I. Medical students from semesters 2 to 6 served as a control group. Questionnaires were used to subjectively analyze the training and support functions of modules I-III.

**Results:**

High participation rates and the fact that the modules were taught numerous times show that doctoral students accept the courses. The objective analysis of module I showed a highly significant knowledge acquisition of the course group (N=55) in contrast to the control group (N=34). The doctoral students rated the course modules I-III with grades between 1.0 and 1.25 (grade A+/A; N=20-65 SD=0-0.44), felt well supported and estimated their learning success as high.

**Conclusion:**

The study indicates knowledge acquisition in module I and a high doctoral student satisfaction with all modules. For an objective analysis of modules II-III, a comparison of completed doctoral theses (course participants vs. non-participants) would be appropriate but would only make sense in a few years. Based on the results of our study, we recommend that other faculties implement similar courses.

## Zusammenfassung

**Hintergrund:**

Obwohl die Mehrheit der Medizinstudierenden in Deutschland promoviert, erfährt nur eine Minderheit eine standardisierte wissenschaftliche Ausbildung, was sich an Qualitätsmängeln medizinischer Promotionsarbeiten äußert. Um Promovierenden der Medizin einerseits eine Unterstützung zu geben und andererseits die Qualität ihrer wissenschaftlichen Arbeiten zu verbessern, wurde das Kursangebot Basics zur medizinischen Dissertation konzeptioniert und wissenschaftlich begleitet.

**Methodik:**

Das Kursangebot besteht aus drei Modulen. Modul I als Grundlagenkurs behandelt neben dem Zeit- und Schreibmanagement, dem Umgang mit Literatur und den Grundsätzen des wissenschaftlichen Arbeitens auch die Kapitelinhalte einer Dissertationsschrift sowie die Präsentation und Verteidigung. Im praktischen Modul II verfassen Promovierende Auszüge von Dissertationskapiteln und erhalten über Peer- und Experten-Begutachtungen Feedback. Modul III umfasst das Training von Promotionsvorträgen und deren Verteidigung. Zur objektiven Analyse wurde ein Multiple Choice Test vor und nach Modul I durchgeführt. Medizinstudierende aus Fachsemester 2 bis 6 dienten als Kontrollgruppe. Anhand von Fragebögen wurden alle Kursmodule I-III hinsichtlich ihrer Ausbildungs- und Unterstützungsfunktion subjektiv analysiert.

**Ergebnisse:**

Hohe Teilnahmezahlen und die vielfache Durchführung der Kursmodule zeigen, dass Promovierende die Kurse akzeptieren. Die objektive Analyse von Modul I ergab einen hoch signifikanten Wissenserwerb der Kursgruppe (N=55) im Gegensatz zur Kontrollgruppe (N=34). Die Promovierenden bewerteten die Kursmodule I-III mit Schulnoten zwischen 1,0 und 1,25 (N=20-65 SD=0-0,44), fühlten sich gut unterstützt und schätzten ihren Lernerfolg als hoch ein.

**Schlussfolgerung:**

Die Studie zeigt eine hohe Promovierenden-Zufriedenheit mit allen Modulen und einen Wissenserwerb durch das Modul I. Zur objektiven Analyse von Modul II-III bietet sich ein Vergleich der fertiggestellten Promotionsarbeiten (Kurs Teilnehmende vs. Nicht-Teilnehmende) an, welcher erst in ein paar Jahren sinnvoll ist. Durch die Ergebnisse unserer Studie empfehlen wir anderen Fakultäten die Implementierung ähnlicher Angebote.

## 1. Introduction

### 1.1. The problem

Between 54 to 70 percent of all medical students successfully complete their doctorates while about one-third of them do not [1], [2], [3], [4]. On the one hand, this indicates a very high willingness to do a doctorate, but on the other, that the doctoral students are often unsuccessful [5], [6]. What is special about the study of medicine is that the doctorate can be started while the medical degree is being pursued. This promises an initial motivation since it saves time, but it often leads to a double burden [5], [7], [8]. Another issue is an insufficient basic scientific education as well as a lack of supervision of doctoral candidates [9]. The quality of medical doctorates is also being criticized at the scientific and socio-political level. Thus, negative catch phrases such as* title research* and *after-work research* reflect the bad reputation of medical doctorates [8].

While there is a high demand for good scientific education by doctoral students and a high demand for quality from the scientific and societal side, there is often a lack of course offerings in this regard. In recent years, the global standards of medical education of the WFME (World Federation for Medical Education), the Medizinstudium 2020 (medical studies 2020) master plan and the Wissenschaftsrat (German council of science and humanities) have called for a strengthening of the scientific education. Individual German medical faculties have responded to this and implemented scientific course concepts [4], [8], [10], [11], [12], [13], [14], [15], [16] as well as quality assurance measures, which were documented in a study of the University Alliance for Young Scientists [17]. While subjective student evaluations are available, objective analyses of such doctoral courses are still lacking [16].

#### 1.2. Initial situation at the medical faculty of the university of Ulm

The official curriculum of the medical faculty of the university of Ulm includes scientific content from the subjects of biometry and epidemiology (semester 7). In addition to evidence-based medicine, various types of research including the planning, methodology and implementation as well as the application of statistical tests are covered. Scientific content is also taught in other events that are included in a longitudinal mosaic curriculum (wise@ulm).

In addition, the University of Ulm offers electives for doctoral students: The experimental medicine course of study introduced in 2005, for example, is a doctoral program for medical students that requires an experimental dissertation. Each year, approximately 35 students are selected with the help of an application and selection process. The support provided consists of professional and scientific supervision, various scientific events, the completion of elective courses and ten months of financial support [18].

The course *Fit für die diss MED* (Fit for the medical dissertation), offered by the communication and information center, is a voluntary course made available to medical students at the university of Ulm. The course, which includes a total of eight hours and is mainly theoretical, covers successful publishing, the scientific framework and the use of computer programs. The content of the medical dissertation chapters is only marginally discussed. 

There is no course offered for doctoral medical students that deals intensively with good scientific practice and the chapter content required for a doctoral thesis. Practical support during the writing process and in preparation for the presentation and defense of a dissertation has been limited as well. Thus, the course “medical dissertation basics: how to write scientific texts and present a doctoral thesis” with a total of three modules (MED I-III) was implemented in 2018, has been taught numerous times since then and has been monitored scientifically.

This raises the following questions:

- Is the Basics MED course with its three modules I-III accepted by students obtaining a doctorate in medicine?
- Can the participation in MED I (module I) result in an acquisition of knowledge by students obtaining a doctorate in medicine?
- How do students obtaining a doctorate in medicine rate the support provided and the scientific content learned during the three modules MED I-III?

## 2. Methods

### 2.1. Course concept 

The course offering “Medical dissertation basics: How to write scientific texts and present a doctoral thesis” (MED I-III) was developed and introduced in 2018. Module I covers scientific fundamentals and teaches the content required for a medical doctoral thesis. Module II teaches students how to write high-quality text. Module III trains students on how to present and defend a doctoral thesis. The sequence of the modules (I → II → III) is based on the chronology of the medical doctoral process and permits students to apply the theoretical content learned (module I) to their own doctorate with the help of practical assignments (module II-III). The course content is based on the official guidelines of the medical faculty of the university of Ulm, observations gathered during the supervision of medical doctoral theses and courses that are already being offered at other universities [9], [11], [15], [16].

#### 2.1.1. Participation information

The course is offered to doctoral students of human and dental medicine. In some cases, students from other degree programs may participate as well. 

Students may take modules I and III as needed. Module I is a prerequisite for module II. The online courses are offered on the Ulm Moodle platform. Modules I and III are offered 3-5 times a year depending on demand while module II is offered throughout the year.

##### 2.1.2. MED I (module I)

Module I is offered to students shortly before or at the beginning of the doctorate program as a one-week online course (nine hours in total). In order to structure the content, eight teaching phases (15 min to 2 hours each) have been defined as either independent study phases or classroom phases (online meetings).

**Figure 1:**
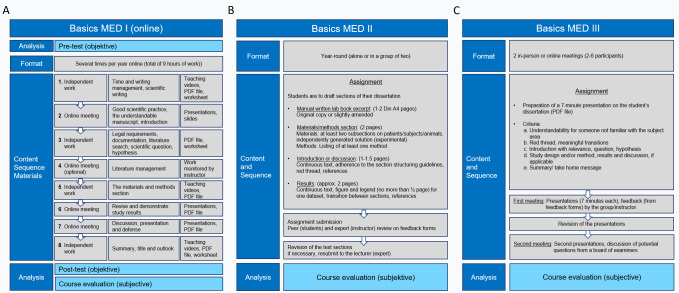
Point A-C: Course concepts with analyses of MED I-III (Module I-III). A. Course organization (phases 1-8), content and materials of MED I, mandatory participation in pre-tests and post-tests (objective analysis), voluntary participation in evaluations (subjective analysis). B. Course organization, sequence and content (assignments with text length) of MED II, voluntary participation in evaluations. C. Course organization, sequence and content of MED III, voluntary participation in evaluations. Abbreviation: MED: Medical Experimental Dissertation Basics.

In the (independent study) phase 1, students are introduced to scientific practice as well as time and writing management with the help of instructional videos, PDF files and worksheets. In the (classroom) phase 2, the instructor lectures on good scientific practice, the development of a comprehensible manuscript and its introduction. The remaining phases cover the legal framework, the scientific question or hypothesis, literature research and management (optional) and the remaining chapters of a dissertation as well as the presentation and defense of a dissertation (see figure 1 (Fig. 1), part A).

##### 2.1.3. MED II (module II)

The online module II is designed for doctoral students who have already taken MED I and have started writing their dissertation. Students may participate individually or as a group of two. The assignments require students to write three to four sections of their own dissertation (see figure 1 (Fig. 1), part B): Excerpt from the laboratory book (writing assignment 1), the materials and methods section (written assignment 2), excerpt of the introduction or discussion (written assignment 3) and excerpt of the results section (written assignment 4). These sections are first subjected to a peer review (feedback from another student) and then to an expert review (from the instructor). For both reviews, a semi-standardized feedback form is used, which was developed by two experts and reviewed by the academic staff members of our working group. If necessary, the doctoral students must submit a revised draft of a given section upon having received their feedback.

##### 2.1.4. MED III (module III)

Module III trains students to present and defend their dissertations. In an individual preparation phase, students prepare a 7-minute presentation of their dissertation and are required to use a brief guideline. The students make their presentations in front of a small group (three to six doctoral students) during a first (online) class. Each presentation is followed by an approximately 30-minute feedback portion (feedback offered by the small group and the instructor) using a customized, semi-standardized feedback form, which was developed in the same manner as the feedback form used in module II. In a revision phase, the presentations are revised and presented again during a second (online) class. Students are provided with further feedback and collect and discuss potential questions such as those that an examination committee might present in order to practice the defense portion of the dissertation (see figure 1 (Fig. 1), part C).

#### 2.2. Study design for the analysis of the course offered (modules I-III)

The MED course study was divided into an objective analysis of the first module and subjective analyses of all modules (I-III).

For the objective analysis of the first module, a multiple choice (MC) knowledge test was developed and used as part of the courses offered from June to October 2020. Since module I was offered three times during this period, there were three test cycles. The test subjects consisted of the participants of module I (course group) and a control group. The selection of the individuals in the control group was subject to the following conditions: They had to be students of human medicine from the semesters 2-6 who had not yet started their doctoral thesis.

The subjective analysis of module I was based on the voluntary student evaluations from June 2020 to July 2021 (N=65). The subjective analyses of module II (N=20) and module III (N=20) were based on the evaluations from 2018 to 2021.

##### 2.2.1. Objective analysis of the knowledge acquisition (module I)

To assess the knowledge acquired due to a participation in MED I (module I), 19 multiple choice questions were developed. In a second step, the test design was reviewed by two experts. Volunteers from our work group (N=7) performed a pretest in a third step [19], [20] and provided feedback about unclear or misleading wording and completion time.

The final test, consisting of eleven A_positive_ type questions (choose one correct answer out of five possible answers) and eight K_Prim_ type questions (choose multiple correct answers out of five possible answers), was administered via the Ulm learning platform Moodle. The knowledge test was administered three days before (pre-test) and three days after (post-test) the course (completion time: max. 20 minutes). Although the same questions were used for the pre-test and post-test, the order of the questions and answers was changed. Participants in the control group were asked to not research the content related to the questions over the course of the study.

With regard to eight K_Prim_ type questions, the number of correct answer options varied (from 2 to 5). If an answer option was correctly selected, one point was awarded so that a maximum of 5 points could be achieved for each K Prim question. Points were deducted for incorrectly selected distractors. The point deduction principle was applied equally to all questions (type A_positive_ and K_Prim_). Consequently, a total score of minus 30 to plus 32 points was possible.

##### 2.2.2. Subjective analysis through student evaluations (modules I-III).

**Figure 2:**
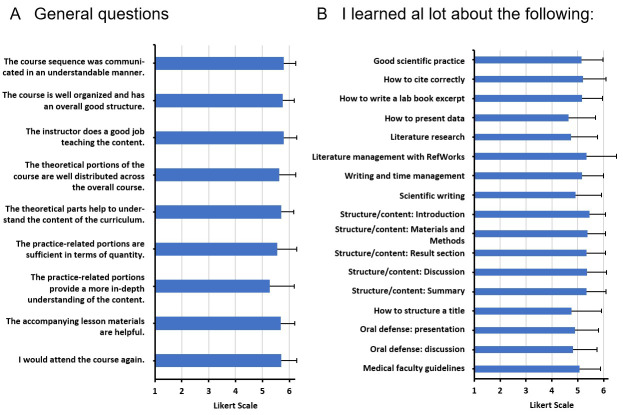
Results from the student evaluations of MED I (Module I). A. General questions about the course. B. Students' assessment of the individually perceived learning success; Likert scale: from 1= "strongly disagree" to 6= "strongly agree". N=65.

**Figure 3:**
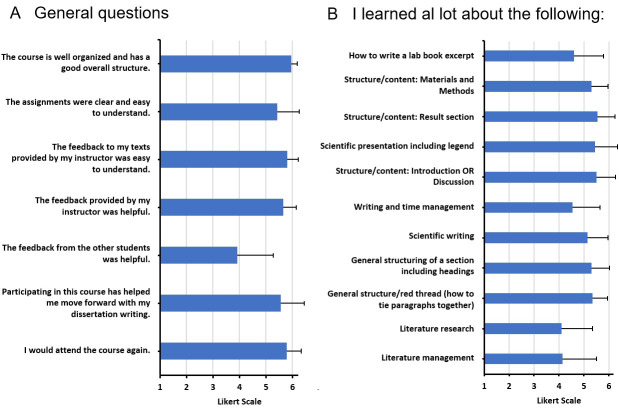
Results from the student evaluations of MED II (Module II). A. General questions about the course. B. Students‘ assessment of the individually perceived learning success; Likert scale: from 1= “strongly disagree” to 6= “strongly agree”. N=20.

**Figure 4:**
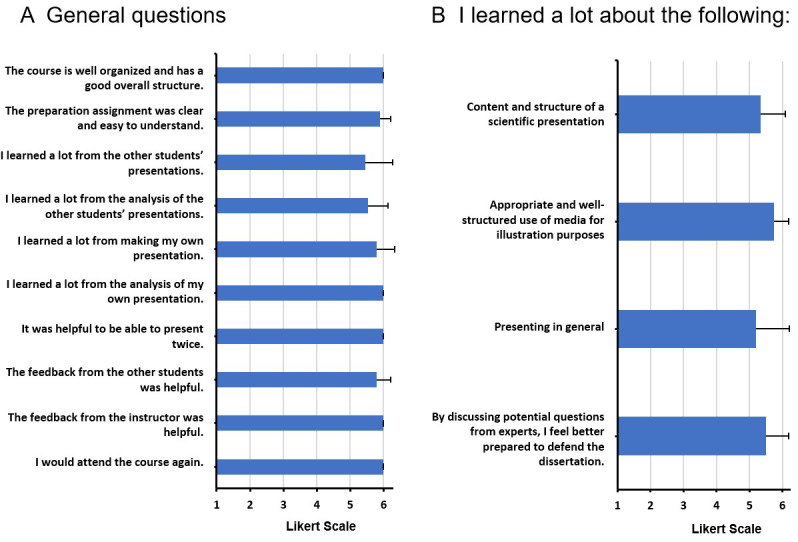
Results from the student evaluations of MED III (Module III). A. General questions about the course. B. Students‘ assessment of the individually perceived learning success; Likert scale: from 1= “strongly disagree” to 6= “strongly agree”. N=20.

For the subjective analysis, semi-standardized questionnaires were developed for all modules. In addition to the socio-demographic data of the participants, data on general and content-related course aspects was collected (e.g., the organization, structure and subjectively perceived learning success; see figure 2 (Fig. 2), figure 3 (Fig. 3) and figure 4 (Fig. 4)), which were assessed with a Likert-type response scale (1=do not agree at all to 6=agree completely). Participants were able to enter praise, criticism or suggestions for improvement in a free text field. The overall module was also evaluated by using a school grade (1=very good, 6=insufficient).

#### 2.3. Data analysis and statistics

All analyses were performed using the SPSS Statistics Version 26 software from the International Business Machines Corporation. For the knowledge test, the total scores of all three test cycles were calculated. The Kolmogorov-Smirnov test did not show a normal distribution of the data, so the nonparametric Wilcoxon signed-rank test for connected samples was used for analysis purposes. An alpha level of 5% was applied. Free-text comments were categorized and quantified according to praise, criticism or suggestion for improvement, following Schneider et al., 2019 [21].

#### 2.4. Ethics

The ethics committee of the University of Ulm did not consider an ethics vote necessary. The participation in the questionnaires and tests was voluntary, anonymous and free of charge. The participants' consent to data processing and data transfer was obtained.

## 3. Results

### 3.1. Participation figures

A total of 171 doctoral students participated in MED I (which was offered six times between July 2020 and November 2021), 21 students participated in MED II (since 2018) and 25 students participated in MED III (which was offered nine times since 2018). The number of participants in the course-related studies was somewhat lower (see figure 1 (Fig. 1) and table 1 (Tab. 1)).

#### 3.2. Objective analysis of MED I

##### 3.2.1. Sociodemographic data of the course and control groups

The socio-demographic data of the course group was obtained from the evaluation forms (section 2.2.2) and data of the control group was based on verbal information provided by the participants.

Of the module I participants, 89% studied human medicine (N=65, see table 1 (Tab. 1)) compared to 100% of control group subjects (N=34). The majority of course participants were female (71%); in the control group, male subjects dominated with 62%. The course participants were on average in semester 7.67 (SD=1.66) while the subjects of the control group were in semester 4.76 (SD=1.35).

##### 3.2.2. Results from the knowledge test

**Figure 5:**
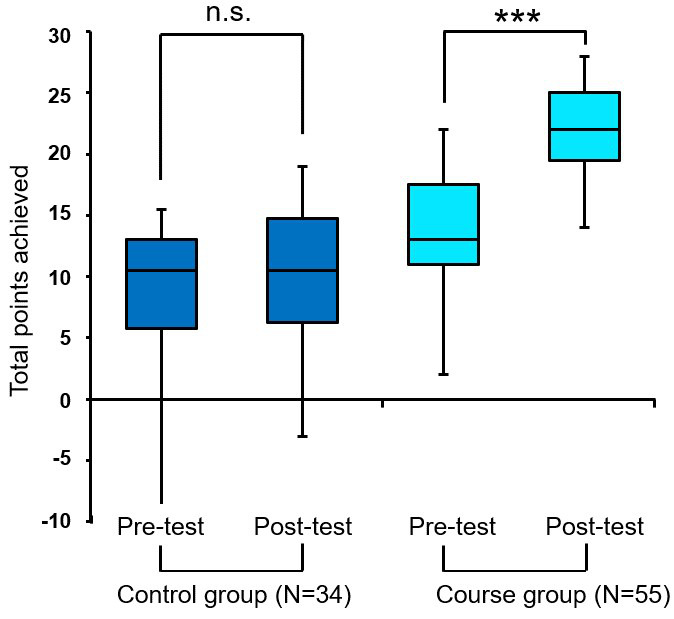
Results from the knowledge test of MED I (Module I) in a boxplot diagram, comparison of test results (pre-test and post-test) of the control group and the course group from three test cycles (June 2020 - October 2020), maximum total score=32 points, negative results possible as well due to a point deduction for wrong answers. Abbreviations: N=subjects of the control group/course group, p-value ***p=0.001; n.s., not significant.

To test for knowledge acquisition in MED I, the results from the pre-test and post-test were compared (see figure 5 (Fig. 5)). The result of the control group remained unchanged with a median of 10.5 points (Q1=5.75 Q3=13) in the pre-test and post-test. Only the dispersion decreased slightly in the post-test. In contrast, the course group showed a significant knowledge acquisition with a median of 13 points in the pre-test (Q1=11 Q3=17.5) and 22 points in the post-test (Q1=19.5 Q3=25) (p<0.001).

#### 3.3. Subjective analyses of MED I-III

##### 3.3.1. Sociodemographic data

The sociodemographic data of the participants (see table 1 (Tab. 1)) shows that the age and semester of study increased from module I to III. Dental and human medical students who had not yet started or had already started their experimental/clinical/retrospective/teaching research participated in Module I. Module groups II and III included human medicine students who were primarily doing experimental work. A large proportion of doctoral students from the experimental medicine student track participated in all modules [18].

##### 3.3.2. Subjective evaluation results

MED I was rated on average with the school grade 1.21 (N=58 SD=0.41), MED II with 1.28 (N=18 SD=0.46) and MED III with the grade 1.0 (N=20 SD=0.00). Additional questions tried to determine how students obtaining a doctorate in medicine assess the support and their learning success in the courses.

##### 3.3.3. Evaluation results for module I

The communication of the general course information (MW=5.80, SD=0.44), the organization and overall structure, and the teaching by the instructor were rated particularly positively. The presentation of data and the literature research (MW=4.74, SD=1.02) scored somewhat worse. The teaching of scientific content such as literature management (MW=5.35, SD=1.16) and the teaching of the chapter content required for a dissertation, led to a subjectively perceived high learning success (see figure 2). Similar results were reflected by the praise expressed in the free text questions in which the course content, the commitment of the instructors and the teaching videos were positively emphasized (see table 2 (Tab. 2)).

##### 3.3.4. Evaluation results for module II

General aspects such as the basic structure, the assignments and the feedback by the instructor (MW=5.80, SD=0.41) were rated good to very good. The peer feedback by fellow students was rated somewhat lower (MW=3.91, SD=1.38). The participants indicated that their writing process had improved (MW=5.55, SD=0.89). Students rated the drafting of the materials and methods section, the introduction or discussion and the results section as particularly instructive and the lab journal entry as (somewhat) instructive (MW=4.60, SD=1.19) (see figure 3 (Fig. 3)). Two students commented on being able to do without the lab book excerpt while others suggested the option of submitting more dissertation sections. The positive comments made up 60% of all comments and included references to the speedy correction and individual feedback provided by the instructor (see table 2 (Tab. 2)).

##### 3.3.5. Evaluation results for module III

MED III, which pertains to the presentation and defense of a dissertation, was characterized by very high student satisfaction. Organizational and structural aspects, the ability to present two times, the analyses and feedback by the instructor were rated very good (MW=6.00, SD=0.00). All students would take the course again (MW=6.00, SD=0.00). Participants rated the learning success pertaining to the general presentation, content and structure of a lecture and the use of media for visualization purposes very highly (see figure 4 (Fig. 4)). In the free texts, the commitment of the instructors in the course design was rated positively. The participants felt that the module provided structure as well as new perspectives and well prepared them for the presentation and defense of their dissertation. Some participants would have liked more basic information on how to give a good presentation (see table 2 (Tab. 2)).

## 4. Discussion

Our study shows that

- all modules of the Basics MED course are accepted by students obtaining a doctorate in medicine.
- participation in MED I (module I) leads to a knowledge acquisition by the students obtaining a doctorate in medicine.
- students obtaining a doctorate in medicine highly rate the support and learning success of scientific content provided in the course modules MED I-III.

### 4.1. Basics MED courses accepted by doctoral students in medicine

At the time the course was implemented, other doctoral programs had already been established at the University of Ulm [18]. Therefore, despite a high demand for doctoral programs throughout Germany, we were interested in whether the course would be accepted [9], [13]. We were able to confirm this based on the number of times the course has been conducted (several times a year) and high participation numbers. The participation figures for Modules II and III were somewhat lower. Possible reasons are that modules II-III become relevant in the later couese of the dissertation (possibly not until later) and the additional time required. For module II, students had to have first completed module I, and continuous texts had to be drafted. In contrast to a scientific term paper (doctoral program at the Charité Berlin), these continuous texts are only excerpts of the student's dissertation, which relativizes the additional effort [15].

#### 4.2. Participation in MED I (Module I) results in knowledge acquisition

To test the degree to which students learned from module I, an MC test was designed and administered before and after the course (pre-test and post-test). It showed a significant knowledge acquisition by the course group compared to the control group. The purpose of the control group was to test for factors that might influence the test results, such as a practice effect due to the test being administered twice [22], and jeopardize their validity. We used identical questions in the pre-test and the post-test and only changed the order, which, according to Golda et al., has no significant influence on the level of difficulty [23].

Due to insignificant differences in the test scores of the control group, a practice effect can be largely ruled out, indicating an objective knowledge acquisition of the course group.

#### 4.3. Doctoral students rate the support and learning success highly

Our subjective analyses show that students considered the basics MED modules I-III as helpful for their doctoral studies. The participants rated the learning gain relating to scientific content high. The learning gain relating to literature research (and management) was insignificantly lower. One reason could be the complexity of the topic, which is difficult to grasp in a 9-hour course. The ability to manage literature is often acquired over a longer period of time, such as the entire doctoral period [13]. In the evaluation of MED II, the feedback by the instructor was rated more helpful than the peer feedback provided by fellow students (see figure 3 (Fig. 3)). Examples from the literature show that students can generally benefit from a feedback culture (including peer feedback) [24], [25]. Doctoral students are at the beginning of their academic career and have yet to develop a critical eye for academic texts. This process is positively supported by the involvement in peer feedback. 

Individual participants rated the relevance of the laboratory book excerpt as low. The Wissenschaftsrat and the instructors believe that this portion of the module is very relevant for ensuring scientific standards [12].

Overall, however, the results at the subjective level are consistent with calls (by the Wissenschaftsrat, WFME, etc.) for more intensive support and scientific training [11], [12]. Studies evaluating other doctoral programs have resulted in similar conclusions [15], [16].

#### 4.4. Limitations

The limiting factor of the knowledge test relating to module I is that only MC questions were used. Unlike open-ended question formats, it is possible that MC questions are answered correctly not due to sound knowledge but rather because students recognize key words [26]. On the other hand, this type of question is commonly used in exams and allows for a standardized and quantitative evaluation [26].

In addition, the course group included students who were on the perennial experimental medicine study track. It is possible, albeit unlikely, that the doctoral program may influence the test results, but this cannot be ruled out. Other limitations include differences in the test groups: The majority of the course participants had already started their doctorate while the control group had not (yet) started. Since many doctoral students of the Medical Faculty had already taken MED I, the number of doctoral students suitable for the control group was limited. Furthermore, there was a lack of data (e.g., e-mail addresses) for a targeted search for subjects. Therefore, we chose medical students from semesters 2-6 who were younger on average and were not yet pursuing their doctorate and with whom we had had contact in other courses. We received more feedback from male subjects, resulting in a different gender distribution between course and control subjects. In addition, the control group did not include any participants from the Experimental Medicine study track. This is due to the fact that almost all of the 35 participants who had just received funding during the study period took part in MED I because the Experimental Medicine study track accepts the MED modules as electives [18].

Another approach to determine whether the knowledge increase was due to the course would be to test content that was not covered in the course. However, additional questions would have led to an increase in processing time, which might have decreased the willingness to participate in the study. 

In addition to uncertain objectivity and validity, another limitation of voluntary evaluations is that they are conducted online [27]. Online evaluations can be perceived as more anonymous than face-to-face surveys [28]. Without a tangible expectation from the instructors present, the response rate may have been lower. Advantages of more anonymous (online) surveys, however, are more honest expressions, especially of criticism, which are valuable for the further development of a course [28], [29].

## 5. Summary and outlook

Our study allows for both an objective and subjective analysis of a course designed to support students obtaining a doctorate in medicine. The MED I-III modules were accepted and evaluated very positively. MED I objectively increased the participants’ knowledge. For an objective analysis of MED II, a grade comparison of the completed dissertation would be conceivable (participants compared to non-participants). Analogously, the success of the presentation and defense of the dissertations could be compared for an objective analysis of MED III. It will take a few years, however, to conduct such case-control studies since there is often a time lag of several years between participation in the course and the completion of the doctorate [5].

Based on our results to date, we recommend that other universities develop similar courses.

## Competing interests

The authors declare that they have no competing interests. 

## Figures and Tables

**Table 1:**
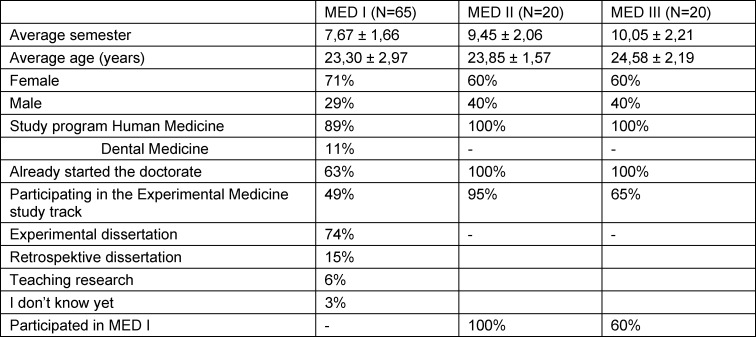
Sociodemographic data from student evaluations of MED I-III in mean +/- standard deviation or in percent %; N=number of evaluators.

**Table 2:**
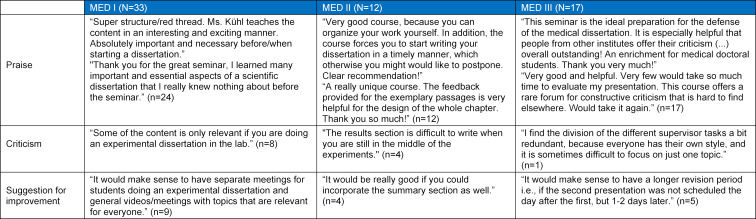
Exemplary free texts for the question: “Criticism/praise/suggestion for improvement” from the student evaluations of MED I-III, N=overall number of free texts, n=number of specific comments (praise/criticism/suggestion for improvement)
